# Prevalence of tuberculosis infection among patients with Takayasu arteritis: a meta-analysis of observational studies

**DOI:** 10.1038/s41598-023-49998-y

**Published:** 2023-12-18

**Authors:** Liping Li, Fang Zhou, Fen Li, Jinwei Chen, Xi Xie

**Affiliations:** 1 Department of Rheumatology, The Second Xiangya Hospital of Central South University, Changsha, China; 2 Clinical Medical Research Center for Systemic Autoimmune Diseases in Hunan Province, Changsha, China

**Keywords:** Autoimmune diseases, Rheumatology, Vasculitis syndromes

## Abstract

To clarify the risk of tuberculosis (TB) infection in patients with Takayasu arteritis (TAK). In this study, we conducted a comprehensive search across multiple databases, including PubMed, Web of Science, Embase, Cochrane, and Medline, from the inception of the Literature Library to May 16, 2023. Using a specific set of keywords, including “Takayasu Arteritis”, “Tuberculosis”, and “Mycobacterium tuberculosis”, the main objective of this search was to identify all relevant observational studies, including case-control studies, cohort studies, and cross-sectional studies, that report the prevalence of TB in individuals diagnosed with TAK. Two independent evaluators rigorously screened the studies, extracted data, and assessed the study quality using the Joanna Briggs Institute (JBI) critical appraisal tools. Statistical analyses were conducted using R software version 4.3.0, which allowed for the synthesis of prevalence and subgroup analyses. Subgroup analyses were stratified based on quality scores, World Health Organization regional categorizations, and TB categories. Assessment of publication bias was performed using a funnel plot. The study included a total of 30 studies with 5548 participants. The findings showed that individuals with TAK exhibited an average prevalence of TB infection at 31.27% (95% CI 20.48–43.11%). Significantly, the prevalence of TB infection demonstrated notable regional disparities, ranging from 16.93% (95% CI 7.71–28.76%) in the Western Pacific Region to 63.58% (95% CI 35.70–87.66%) in the African Region. Moreover, the study revealed that patients with TAK displayed a high prevalence of latent TB infection (LTBI) at 50.01% (95% CI 31.25–68.77%) and active TB at 14.40% (95% CI 9.03–20.68%). The high heterogeneity observed in the data highlights significant variability in TB infection rates among the populations studied, with the African Region exhibiting the highest rates. The study concludes that there is a high prevalence of TB infection in the TAK population, with regional variations. Consideration should be given to  implementing rigorous TB screening measures and preventive interventions specifically tailored for the TAK population.

## Introduction

Takayasu arteritis (TAK) is a chronic granulomatous vasculitis that primarily affects the large-sized arteries, especially the aorta and its major branches^1^. The etiology of TAK remains unclear, but preliminary studies suggest the potential involvement of Mycobacterium tuberculosis (MTB)^2^. Epidemiological observations reveal a higher prevalence of TAK in Eastern European countries, India, Japan, Asia, and South America^3,4^. These regions are characterized by an increased prevalence of tuberculosis (TB). Both TAK and TB share the commonality of presenting with granulomatous lesions, and the co-occurrence of TAK and TB has been consistently documented in several case reports^5–7^, prompting speculation for decades regarding a potential interconnection^8,9^. Nevertheless, a comprehensive and objective scrutiny of this association is still lacking. Consequently, there is a compelling need for further research to explore and clarify this potential correlation.

The main treatment options for TAK include glucocorticoids, immunosuppressive agents, and biological agents^10,11^. However, these treatments may lead to the spread of MTB or the activation of latent TB. Conversely, TB has been implicated in instigating the formation of saccular aneurysms, exacerbating symptoms of TAK, and making its management more complex^12,13^. Hence, it is crucial to conduct systematic and proactive TB case detection within this group. Nonetheless, there is still a lack of comprehensive data regarding TB infection in this population. A comprehensive global assessment of the TB burden in this population could support policy development, strengthen efforts for TB prevention and control, and provide recommendations for future management of the TAK population. The principal aim of this study was to investigate the prevalence of TB infection among individuals with TAK.

## Methods

### Search strategy

An extensive search was conducted across various databases, including PubMed, Web of Science, Embase, Cochrane, and Medline, covering the inception of the Literature Library up to 16 May 2023. The search utilized a narrowed down set of terms, namely "Takayasu Arteritis", "Tuberculosis", and "Mycobacterium tuberculosis", with a language restriction to English. Additionally, relevant literature was also collected through retrospective references. (The search strategy is provided in Appendix Tables A to E).

### Inclusion criteria and exclusion criteria

Inclusion Criteria: (1) The study population must consist of individuals who meet either the 1990 American College of Rheumatology^14^ or the 2022 American College of Rheumatology/EULAR classification criteria for TAK^15^. Studies lacking explicit mention of diagnostic criteria were also included in our analysis, as long as the diagnosis of TAK in the study population was clearly specified. (2) The literature should specifically describe cases of TB infection in this patient population. (3) Acceptable study types include cross-sectional studies, cohort studies, case–control studies, and other observational studies.

Exclusion criteria: (1) duplicate publications or literature employing identical methods; (2) literature in which the study population explicitly excluded TB; (3) literature lacking valid data; (4) literature with ambiguous or incomplete data; (5) cases of TB infection during treatment with biological agents.

### Study selection and data extraction

Two independent reviewers, Liping Li and Fang Zhou, scrutinized the titles and abstracts of all articles retrieved through the search strategy. Conference abstracts and dissertations were also included if they met the specified criteria. In cases where there were conflicting assessments, resolution was achieved through discussion with the corresponding author, Xi Xie. The data extracted from the included studies included key information such as the first author, publication year, study location, study design, sample size, TB category, number of active TB cases and latent TB infections, and TB burden category of the country being studied. Comprehensive data synthesis was conducted using Microsoft Excel 2016 (Table 1).

**Table 1:** Characteristics of the individual studies on the prevalence of TB among patients with TAK included in the current meta-analysis.

Author year	Country	WHO region category	Study period	Study setting	TB diagnostic method	Sample size	TB cases	Age	TB category	quality score
Joseph^20^	India	South-East Asia Region	1996–2022	Single-center study	–	942	64	28 (21–38)	ATB	High
Cilliers^21^	African	Africa Region	1993–2015	multicenter study	PPD	55	40	9.7 ± 3.04	39LTBI,1ATB	High
Liao^22^	China	Western Pacific Region	2013–2021	Single-center study	–	141	19	39 ± 12	ATB	High
Luo^23^	America	Region of the Americas	–	Single-center study	medical history and/or QTB	80	8	not available	8LTBI,2ATB	Medium
Zhou^24^	China	Western Pacific Region	2008–2021	Single-center study	–	1789	30	not available	ATB	High
Pedreira^25^	Brazil	Region of the Americas	2018–2018	multicenter study	QTB	22	8	36.5(32–50)	LTBI	High
Zhang^9^	China	Western Pacific Region	1992–2017	Single-center study	–	1105	109	27.6 ± 10.0	ATB	High
Negalur^26^	India	South-East Asia Region	2016–2017	Single-center study	QTB and/or MT	46	15	36.9	LTBI	Medium
Clemente^27^	Brazil	Region of the Americas	1988–2012	multicenter study	Tuberculin skin test	61	13	9.2 ± 4.2	12LTBI,13ATB	High
Sahin^28^	Turkey	European Region	2002–2017	Single-center study	Tuberculin skin test	16	4	10.9 ± 4.8	ATB	Medium
Kumar^29^	India	South-East Asia Region	–	Single-center study	–	100	6	14	ATB	Medium
Aeschlimann^30^	Canada	Region of the Americas	1986–2015	Single-center study	–	27	3	12.4 (9.1–14.4)	ATB	High
Zhiping^31^	China	Western Pacific Region	2003–2015	Single-center study	-	82	38	Childrens	ATB	Low
Mastrocinque^32^	Brazil	Region of the Americas	–	Single-center study	Mantoux test	14	3	9.4	LTBI	Low
Lin^33^	Korea	Western Pacific Region	1994–2014	Single-center study	–	267	47	31.35 ± 9.56	ATB	High
Fifi-Mah A^34^	African	Africa Region	2001–2011	multicenter study	–	9	4	29	ATB OR LTBI	Low
Karadag^35^	Turkey	European Region	–	multicenter study	QTB	94	6	40	55LTBI,6ATB	High
Panico^36^	Brazil	Region of the Americas	1977–2006	Single-center study	–	36	3	31.7 (± 13.7)	ATB	High
Mwipatayi^37^	Australia	Western Pacific Region	1952–2002	Single-center study	–	272	54	25	ATB	High
Robles^38^	Mexico	Region of the Americas	–	Single-center study	Tuberculin skin test	44	8	35	35LTBI,8ATB	High
Lupi-Herrera^39^	Mexico	Region of the Americas	1955–1974	Single-center study	Mantoux test	107	51	11–30	ATB	Medium
Sharma^40^	India	South-East Asia Region	1982–1997	Single-center study	–	10	4	22.6 ± 10.2	ATB	High
Arnau^41^	France	European Region	–	Single-center study	–	10	0	42	–	High
Yadav^42^	India	South-East Asia Region	2005–2006	Single-center study	Mantoux test	25	5	21.5	LTBI	High
Francès^43^	France	European Region	1965–1989	multicenter study	–	10	1	-	ATB	High
Chopra^44^	India	South-East Asia Region	–	Single-center study	–	14	3	11–65	ATB	High
Nakao^45^	Tokyo	Western Pacific Region	1959–1965	multicenter study	–	84	22	11–48	ATB	High
Carvalho^46^	Brazil	Region of the Americas	–	Single-center study	mycobacterial DNA	10	6	35.2	LTBI	High
Soto^47^	Mexico	Region of the Americas	–	Single-center study	PPD	40	37	9–41	LTBI	High
Aggarwal^8^	India	South-East Asia Region	–	Single-center study	Mycobacterial antigen	36	33	22.8	LTBI	High

TB: tuberculosis, ATB: Active tuberculosis, LTBI: Latent tuberculosis infection, –: Not available, PPD: purified protein derivative test, QTB: QuantiFERON-TB Gold Plus test.

### Literature quality assessment

The evaluation of study quality adhered to the Joanna Briggs Institute (JBI) critical appraisal tools^16^, with outcomes stratified into low, medium, and high grades if the quality scores were < 60%, 60% to 80%, and > 80%. Two independent investigators, Liping Li and Fang Zhou, assessed the quality of the study, and the corresponding author, Xi Xie, resolved any inconsistencies.

### Data synthesis and statistical analysis

The dataset was exported to R statistical software version 4.3.0 for thorough statistical analysis. An estimate of the pooled TB prevalence in patients with TAK was calculated, along with a 95% confidence interval (CI). The random-effects model was employed, considering significant heterogeneity among studies. Subgroup analyses were conducted based on different categories. Heterogeneity among studies was evaluated using the I2 heterogeneity test, with a value above 50% indicating substantial heterogeneity^17,18^.

### Definition of the terms

The diagnosis of LTBI relies on either a positive tuberculin skin test (TST) or an interferon-gamma release assay (IGRA). Active TB was diagnosed based on a comprehensive assessment that included evaluating TB symptoms (such as cough, sputum, hemoptysis, fever, and chest pain lasting for over two weeks), conducting chest radiography, and performing bacteriological examination^19^. The latter included a positive culture of the acid alcohol-resistant bacillus (BAAR), a positive polymerase chain reaction (PCR) test, confirmation through culture or identification of Mycobacterium tuberculosis in tissues or body fluids, or a histopathological finding compatible with caseous granuloma. While some literature did not explicitly delineate TB diagnostic methodologies, it unequivocally specified the presence of either active or latent TB.

## Results

### Basic characteristics of the included literature and quality evaluation

**Figure 1:**
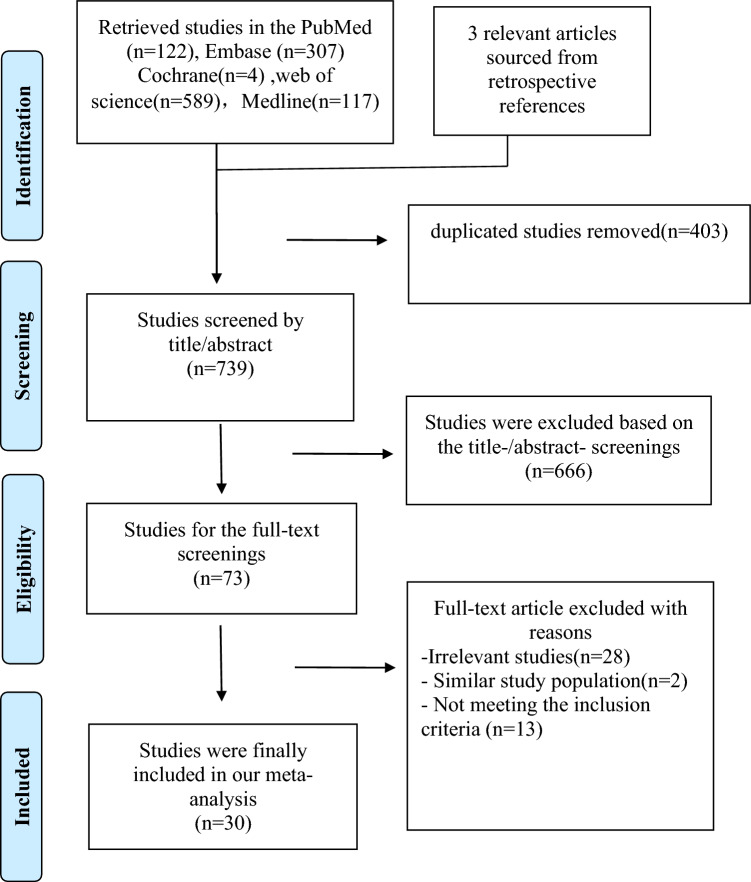
PRISMA flowchart for screening and selection of studies for a meta-analysis of TB among patients with TAK.

The schematic depiction of the literature selection process is shown in Fig. 1. A total of 1139 original articles were retrieved through electronic databases, with an additional three relevant articles sourced from retrospective references. Following rigorous adherence to predefined inclusion criteria, a final selection was made, comprising 30 eligible articles. The individual study sample sizes ranged from 9 to 1789, with data collected from 5548 patients with TAK. Among these patients, TB was detected in 793 individuals. Basic characteristics of the included studies are summarized in Table 1. Predominantly, the studies were geographically concentrated in India (six), Brazil (six), China (four), Mexico (three), Africa (two), Turkey (two), France (two), the USA (one), Canada (one), Korea (one), Australia (one), and Japan (one), with a relatively higher concentration in India, China, and Brazil. Regarding quality, 22 studies were rated as high-quality, while 5 studies were rated as medium quality.

### The overall prevalence of TB infection among patients with TAK

**Figure 2:**
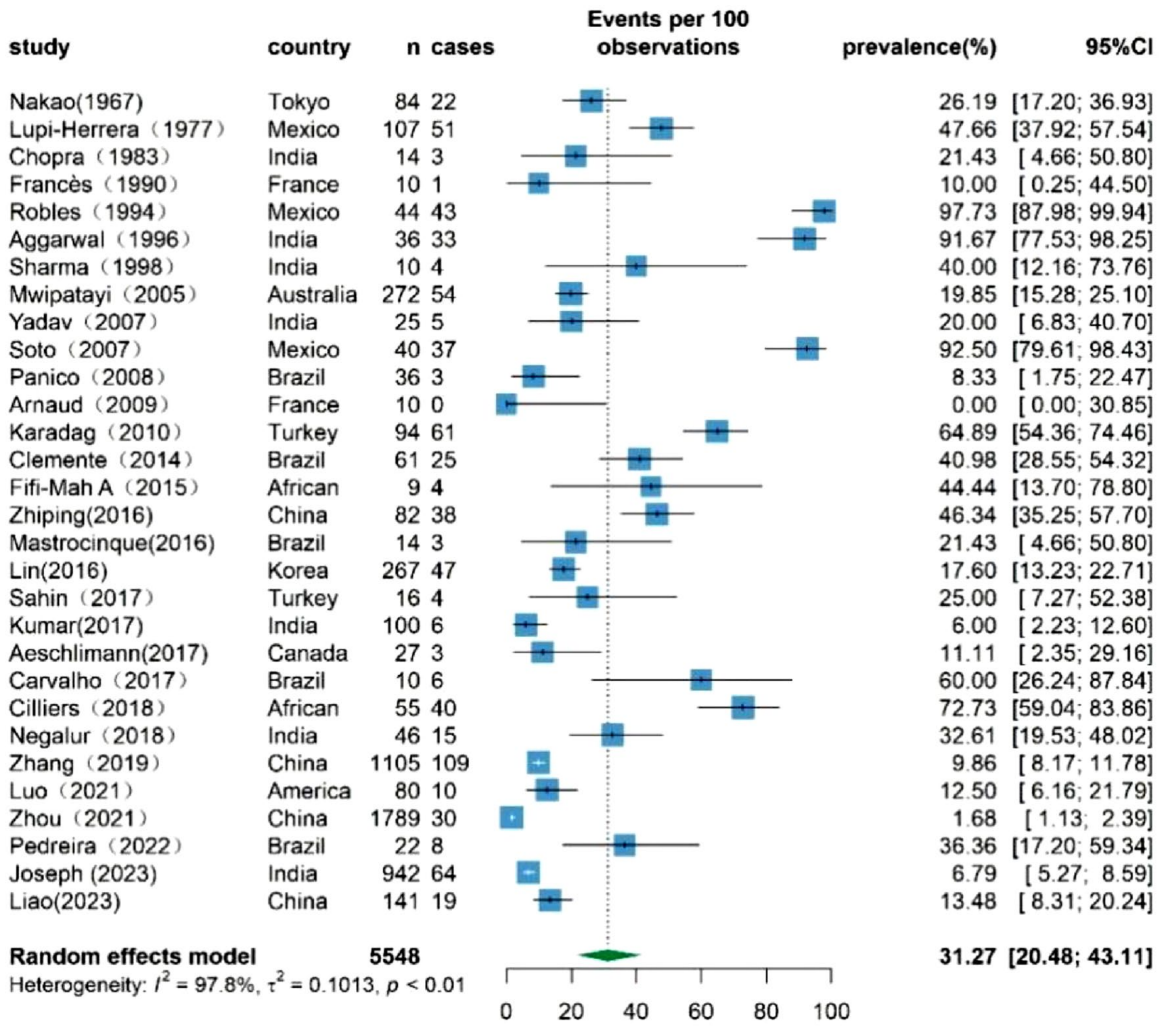
Forest plot showing the prevalence of Mycobacterium TB infection among TAK patients, with 95% CI.

A comprehensive assessment involving 30 studies reported the prevalence of TB infection among patients with TAK. The heterogeneity test revealed significant variation among studies (I^2^ = 97.8%,* p* < 0.01), which required the use of a random-effects model for analysis. The results showed a significant pooled TB infection rate of 31.27% (95% CI 20.48–43.11%) among patients with TAK. This rate surpasses the globally reported average TB infection rate in 2022, which stands at 0.134% in the general population^48^. Figure 2 eloquently presents the summary of TB infection rates among patients with TAK.

### The prevalence of TB infection among TAK patients based on WHO regional categories

**Figure 3:**
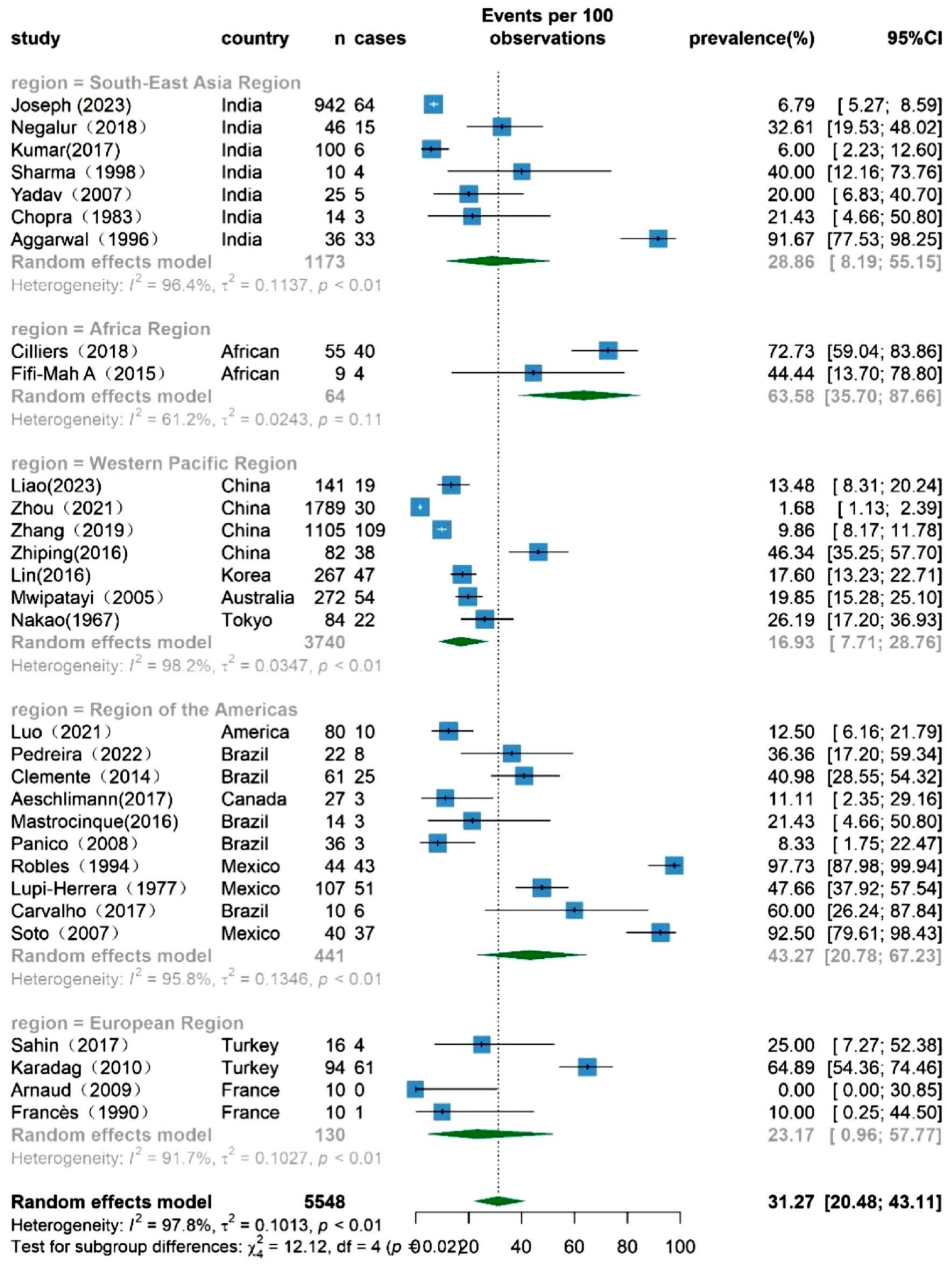
Forest plot of the subgroup analysis based on WHO regional categories, showing the prevalence of TB infection among TAK patients, with 95% CI.

In order to provide a comprehensive understanding of TB prevalence among TAK patients in different geographical regions, a thorough stratified meta-analysis was conducted using WHO regional categories (Fig. 3). Seven studies originated from countries within the South-East Asia Region, two from the Africa Region, seven from the Western Pacific Region, ten from the Region of the Americas, and six from the European Region, with no representation from the Eastern Mediterranean Region. Furthermore, 15 studies were reported from countries categorized as having a high TB burden based on the 2022 Global TB Report. The results revealed that the prevalence of TB in TAK patients was 28.86% (95% CI 8.19–55.15%) in the South-East Asia Region, 63.58% (95% CI 35.70–87.66%) in the Africa Region, 16.93% (95%CI 7.71–28.76%) in the Western Pacific Region, 43.27% (95% CI 20.78–67.23%) in the Region of the Americas, 23.17% (95% CI 0.96–57.77%) in the European Region. Notably, there was a high level of heterogeneity that persisted across these regional estimates.

### The prevalence of TB infection among patients with TAK based on TB category

**Figure 4:**
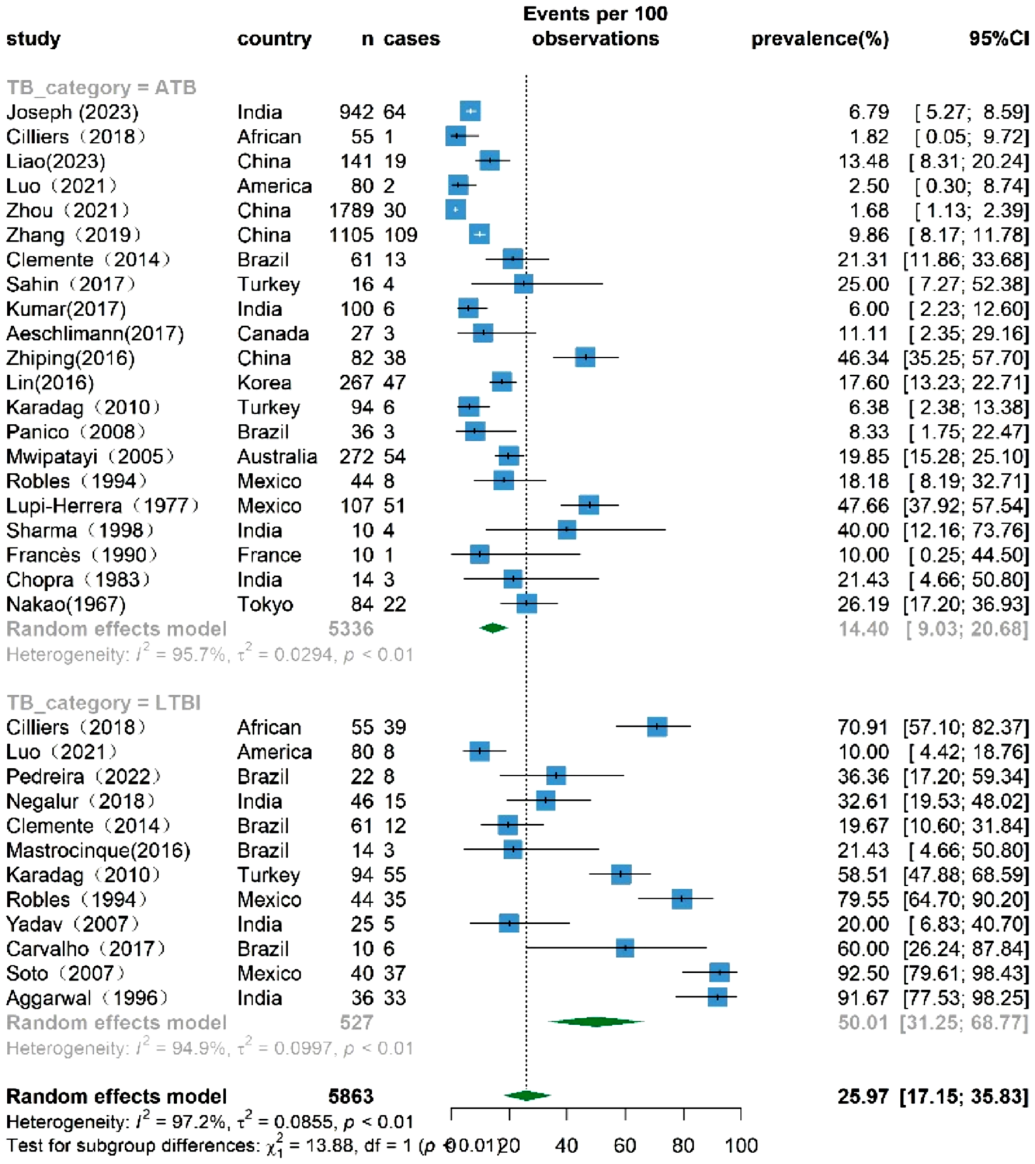
Forest plot of the subgroup analysis based on TB category, showing the prevalence of TB infection among TAK patients, with 95% CI.

Conducting a subgroup analysis based on TB category, the findings are visually presented in Fig. 4. Ultimately, 28 studies were included in the analysis, as two studies did not specify the type of TB. The results showed a prevalence of ATB at 14.40% (95% CI 9.03–20.68%) and LTBI at 50.01% (95% CI 31.25–68.77%). Heterogeneity remained high with I2 values of 95.7% and 94.9% in the ATB and LTBI groups, respectively.

### The prevalence of TB infection among TAK patients based on the quality assessment

**Figure 5:**
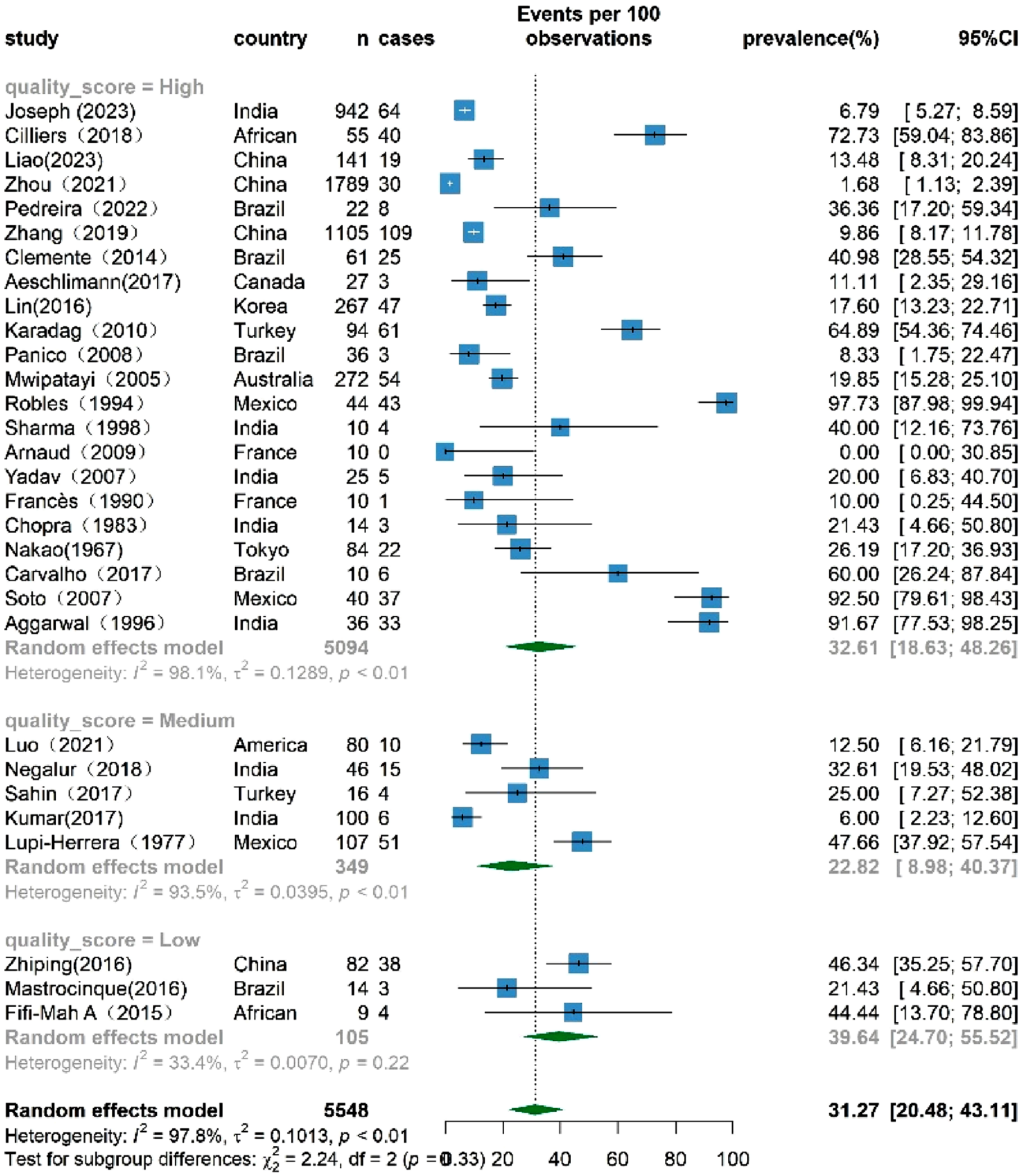
Forest plot of the subgroup analysis based on quality score, showing the prevalence of TB infection among TAK patients, with 95% CI.

The results of the quality assessment are visually presented in the forest plot shown in Fig. 5. The prevalence, stratified by study quality, was 32.61% (95% CI 18.63–48.26%) for high-quality studies and 22.82% (95% CI 8.89–40.37%) for those rated as medium quality. The difference in prevalence is not statistically significant because the 95% CIs overlap, indicating a possible random variation in any observed discrepancy. Conversely, only three low-quality conference abstracts reported a prevalence of 39.64% (95% CI 24.70–5.52%). High heterogeneity was evident, with I2 values reaching 98.1% and 93.5% in the high and medium groups, respectively.

### Sensitivity analysis

**Figure 6:**
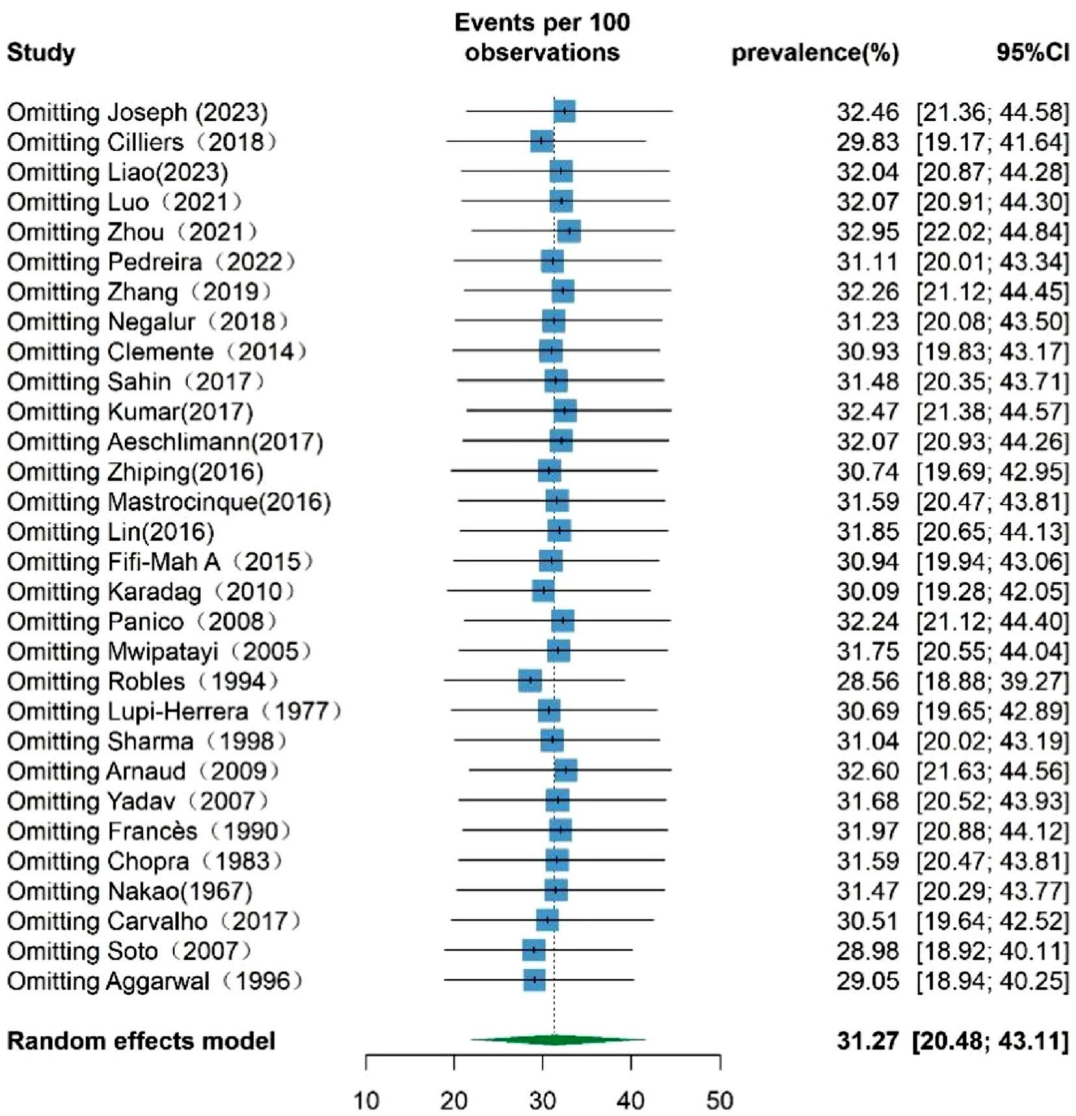
Leave-1-out sensitivity analysis of the influence of a single study on the pooled prevalence of TB infection among TAK patients.

As revealed by the leave-1-out sensitivity analysis, illustrated in Fig. 6, the pooled prevalence of TB infection among patients with TAK exhibited a range from 28.56% (95% CI, 18.88–39.27%) to 32.95% (95% CI 22.02–44.84%) when systematically removing each study. No statistically significant alterations in the pooled prevalence were identified upon the iterative removal of any single study.

### Assessment of publication bias

**Figure 7:**
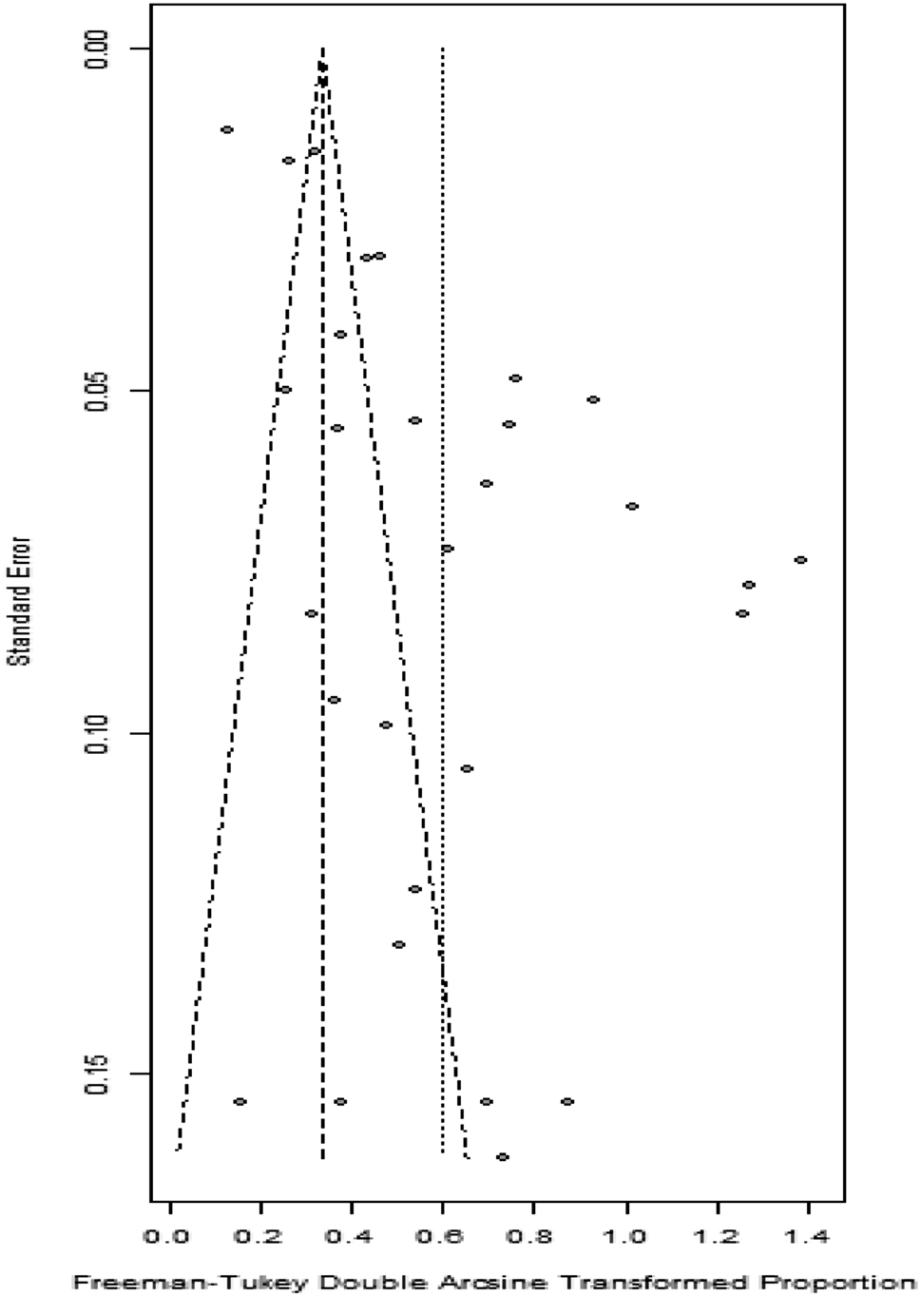
Publication bias of studies on the prevalence of TB infection among patients with TAK.

Visual inspection of the funnel plot revealed significant asymmetry (Fig. 7). Evidence of substantial publication bias was identified using Peters' test (P = 0.028).

## Discussion

### Main findings

The meta-analysis reveals that TB infection among TAK patients is not an unusual phenomenon, exhibiting a prevalence of 31.27%, significantly surpassing the general population rate of 0.134% (Global Tuberculosis Report 2022, who.int).

### Heterogeneity in the literature

Considerable heterogeneity in prevalence was observed across the studies. In an effort to determine its origins, we conducted subgroup analyses based on the burden of TB, TB category, and study quality. Regrettably, these analyses did not explain the observed heterogeneity. Unfortunately, limitations in the data hindered our ability to further analyze the impact of the TB diagnostic method on the observed heterogeneity.

### Strengths and potential limitations

This is the first comprehensive systematic review and meta-analysis of global TB infection rates among patients with TAK, based on TB burden. The analysis includes separate evaluations for active and latent TB infection. Prior systematic reviews summarizing the association between TAK and TB have shown similar results to our study, with both revealing a high risk of TB infection in the TAK population^49,50^. Nevertheless, previous research has not provided a comprehensive summary of TB infection prevalence among TAK patients nor has it conducted subgroup analysis based on the background of TB burden. In contrast, our study not only summarizes the prevalence of TB infection among TAK patients but also provides a comprehensive breakdown of infection rates based on TB burden.

But the interpretation of our findings should be approached with careful consideration of several noteworthy limitations in this systematic review and meta-analysis. Firstly, a limitation arises from the relatively limited inclusion of clinical articles, as none of the 30 studies included in the analysis originated from the Eastern Mediterranean Region. This could undermine the credibility of the original results from the included studies. Secondly, the investigation into the incidence of TB infection among TAK patients encounters constraints due to the low prevalence of TAK in certain countries. Consequently, cases of TB infection in TAK patients are rare, often limited to case reports, which may potentially affect the outcomes of meta-analyses. Thirdly, there is clinical heterogeneity among studies, particularly due to the divergent methods used in diagnosing TB. Although TB diagnostic methods were described in 17 studies, the lack of standardization complicates subgroup analysis. Studies did not impose restrictions on TB testing methods, leading to inconsistent accuracy and sensitivity of TB testing devices, which in turn affects the interpretation of results. Fourthly, the results are limited by significant study heterogeneity, and future investigations should aim to better understand this heterogeneity by analyzing factors such as TB testing methods. Consequently, validating or refuting our results will require additional large-scale clinical studies in the future. Finally, it is imperative to acknowledge that this study primarily relies on English literature, which may introduce selection bias and overlook relevant studies. As a result, the accuracy of value estimations may be affected.

### Clinical implications

Our study showed that the prevalence of TB infection among patients with TAK was 31.27%. Out of these patients, 14.40% were diagnosed with ATB, while a concerning 50.01% were diagnosed with LTBI. This indicates a significant potential for TB development within the TAK population. Two potential contributing factors emerge. Firstly, TAK exhibits a higher prevalence in populations with high rates of TB infection. Furthermore, previous evidence suggests that TB infection may act as a trigger for the development of TAK^51^. The arterial pathology of TAK showcases granulomatous lesions, similar to those observed in TB. However, additional comprehensive studies are required to verify any direct correlation between TB and TAK^2^.

Several studies have elucidated that Mycobacterium tuberculosis-associated proteins are highly expressed in patients with TAK, including the 65-KD heat-shock protein (HSP65)^52,53^. Additionally, Soto^54^ found that the S6110 and HupB genes of MTB were present in the arterial tissue of 70% of TAK patients, indicating that Mycobacterium TB infection may significantly contribute to arterial injury in TAK. Cross-reactive antigens that mimic MTB may be present in the arterial walls of patients with TAK^55^. However, there is currently no direct evidence linking Mycobacterium tuberculosis to arterial injury in TAK. Some studies do not support the association of TB with TAK^41^, and it is unclear whether TAK increases the risk of TB infection.

TB constitutes a significant public health issue globally due to its high transmission rates within the population. ATB poses a significant infection risk, especially for individuals with compromised immune systems^48^. Patients with rheumatic immune diseases are at a higher risk of contracting TB, particularly those using immunosuppression^56,57^. The treatment of TAK mainly consists of glucocorticoids, immunosuppressive agents, and biological agents. However, these treatments may increase the risk of TB infection. Therefore, it is recommended to screen patients for evidence of TB infection prior to initiating treatment. For patients with LTBI, aggressive prophylactic anti-TB treatment is recommended, especially in countries with a high burden of TB.

## Conclusions

In conclusion, our study revealed a population of individuals with TAK who had a high TB infection rate of 31.2%, which was particularly prominent in the African region. The WHO manual for TB planning prioritizes the detection of cases and preventive interventions in high-risk populations (e.g., household contacts of individuals with confirmed tuberculosis), but neglects individuals with rheumatic immune diseases like TAK. This study emphasizes the significance of TB screening in the TAK population for future clinical practice.

## Data Availability

All data generated or analyzed during this study are included in this published article and its supplementary information files.
